# Acute-Onset Visual Impairment in Wilson's Disease: A Case Report and Literature Review

**DOI:** 10.3389/fneur.2022.911882

**Published:** 2022-06-14

**Authors:** Zi-Wei Zheng, Meng-Hui Xu, Chuan-Bin Sun, Zhi-Ying Wu, Yi Dong

**Affiliations:** ^1^Department of Neurology and Department of Medical Genetics in Second Affiliated Hospital, Key Laboratory of Medical Neurobiology of Zhejiang Province, Zhejiang University School of Medicine, Hangzhou, China; ^2^Eye Center, Second Affiliated Hospital of Zhejiang University School of Medicine, Hangzhou, China

**Keywords:** visual impairment, Wilson's disease (WD), copper, iron, *ATP7B*

## Abstract

**Objective:**

We reported the case of a patient with Wilson's disease (WD) with acute-onset visual impairment and summarized previously reported cases to make physicians aware of the complicated clinical expressions of WD and improve diagnosis efficiency.

**Methods:**

The patient was recruited from the Second Affiliated Hospital of Zhejiang University School of Medicine. Clinical data, including cranial images, laboratory tests, and ophthalmic findings were obtained. The PubMed database was searched for published cases of WD with visual impairment.

**Results:**

We reported a 22-year-old male who presented with hand tremor, personality change, and acute-onset binocular vision blurring. WD was considered to be closely correlated with neuropsychiatric and ocular involvements. After low-copper diet and regular copper-chelation therapy, the related symptoms improved compared to before. Six WD cases of optic neuropathy have been reported, including ours. The patients usually had neurological and/or hepatic symptoms for a period without any treatment. All the reported cases manifested as acute episodes of visual changes, and the ocular manifestations improved after copper-chelation treatment.

**Conclusions:**

Excess copper accumulation may be a rare cause of visual impairment in patients with WD. While the etiology behind patients' acute-onset visual impairment remained uncertain, the possibility of WD should be considered through neuropsychiatric and hepatic symptoms, corneal K-F rings, decreased serum ceruloplasmin, and low likelihood or exclusion of other causes. Clinicians need to recognize this rare manifestation and give appropriate treatment to avoid misdiagnosis and unnecessary overtreatment.

## Introduction

Wilson's disease (WD), also known as hepatolenticular degeneration, is an autosomal recessive disorder of copper metabolism and is caused by homozygous or compound heterozygous mutations in *ATP7B* (1). Pathogenetic variants in *ATP7B* result in impaired biliary copper excretion and decreased serum ceruloplasmin, leading to excess copper deposition in the brain, liver, kidney, bones, cornea, and other tissues and organs. Therefore, the clinical manifestations of WD are complex and variable, and early diagnosis can be challenging (2). However, WD is currently one of the few treatable neurogenetic diseases, emphasizing the importance of timely diagnosis. With standardized treatment, the prognosis of patients can be promising. In general, early diagnosis and standardized treatment of WD are crucial.

The most typical ophthalmological sign is the Kayser-Fleischer (K-F) ring, which is formed by copper deposition in Descemet's membrane in peripheral cornea (3), and was detected in 97.6% of patients with neurologic symptoms (4). Sunflower cataract, which accounts for ~1.2–17% of patients with WD (5), is due to copper deposition under the lens capsule. Notably, the K-F ring and sunflower cataract generally do not induce impaired vision and can disappear after copper chelation therapy (6–10).

We reported the case of a 22-year-old male who presented with hand tremor, personality change, and acute-onset binocular vision blurring. After low-copper diet and regular copper-chelation therapy, the neuropsychiatric and ocular symptoms have improved compared to before. WD was found to be the cause of his neuropsychiatric manifestation and decreased vision. Besides, we summarized previously reported cases to make clinicians aware of the rare manifestations of WD and improve diagnosis efficiency.

## Methods

### Subject and Genetic Analysis

The patient was recruited from the Second Affiliated Hospital of Zhejiang University School of Medicine. Clinical data, including cranial image, laboratory tests, and ophthalmic findings were obtained. Genomic DNA was isolated from peripheral EDTA-treated blood with a DNA isolation kit (Qiagen Inc, Valencia, CA, United States). Whole exome sequencing (WES) was performed on an Illumina HiSeq X Ten platform (XY Biotechnology Co. Ltd., Hangzhou, China). Sanger sequencing was performed on an ABI 3500xL Dx Genetic Analyzer (Applied Biosystems) to validate the variants, and the procedure was described previously (11). The study was approved by the corresponding ethics committee of the local hospital, and informed consent was obtained from the patient.

### Literature Review Design

The PubMed database was searched for published cases of WD-induced visual impairment. The keywords used were Wilson's disease and visual impairment. All cases or articles written in English were reviewed. Cases in French and German were also extended by reviewing the reference of included published articles. Patient descriptions and characteristics of six cases (including this case) are summarized in Table 1.

**Table 1:** Previously reported cases of Wilson's disease (WD) with acute-onset visual impairment.

**Gender/age**	**Age at onset**	**Onset form**	**Ceruloplasmin mg/L (200–600)**	**Ophthalmologic Findings**	**Neurologic Findings**	**Hepatic Findings**	**References**
M/35	N. A	N. A	N. A	Bilateral optic atrophy	Dysphagia, gait disturbance	Cirrhosis	Soderbergh (12)
M/22	N. A	N. A	N. A	Neither retinal changes nor great visual impairment has been described	N. A	N. A	Rossa (13)
M/46	46	Hepatic	190	The initial deterioration in color vision was followed by a reduction in visual acuity, VER were grossly abnormal	/	Cirrhosis, hepatosplenomegaly, several spider nevi, abnormal liver biochemistry	Gow (14)
M/14	N. A	Neurologic	N. A	N. A	Tremor and slurred speech	End stage of liver disease	Rukunuzzaman et al. (15)
F/20	20	Hepatic	170	Positive APD of the right eye;decreased bedside visual acuity of 20/400 (PH J16) OD	/	Liver biochemistry was abnormal;	Chou et al. (16)
M/22	18	Neurologic	54-62	Decreased visual acuity; abnormal OCT	Involuntary tremor in hands; dysarthria; personality change; gait disturbance	Cirrhosis; splenomegaly	This case

*N. A, not available; VER, visual-evoked response; APD, afferent pupillary defect; OCT, optical coherence tomography*.

## Results

### Case Report

**Figure 1:**
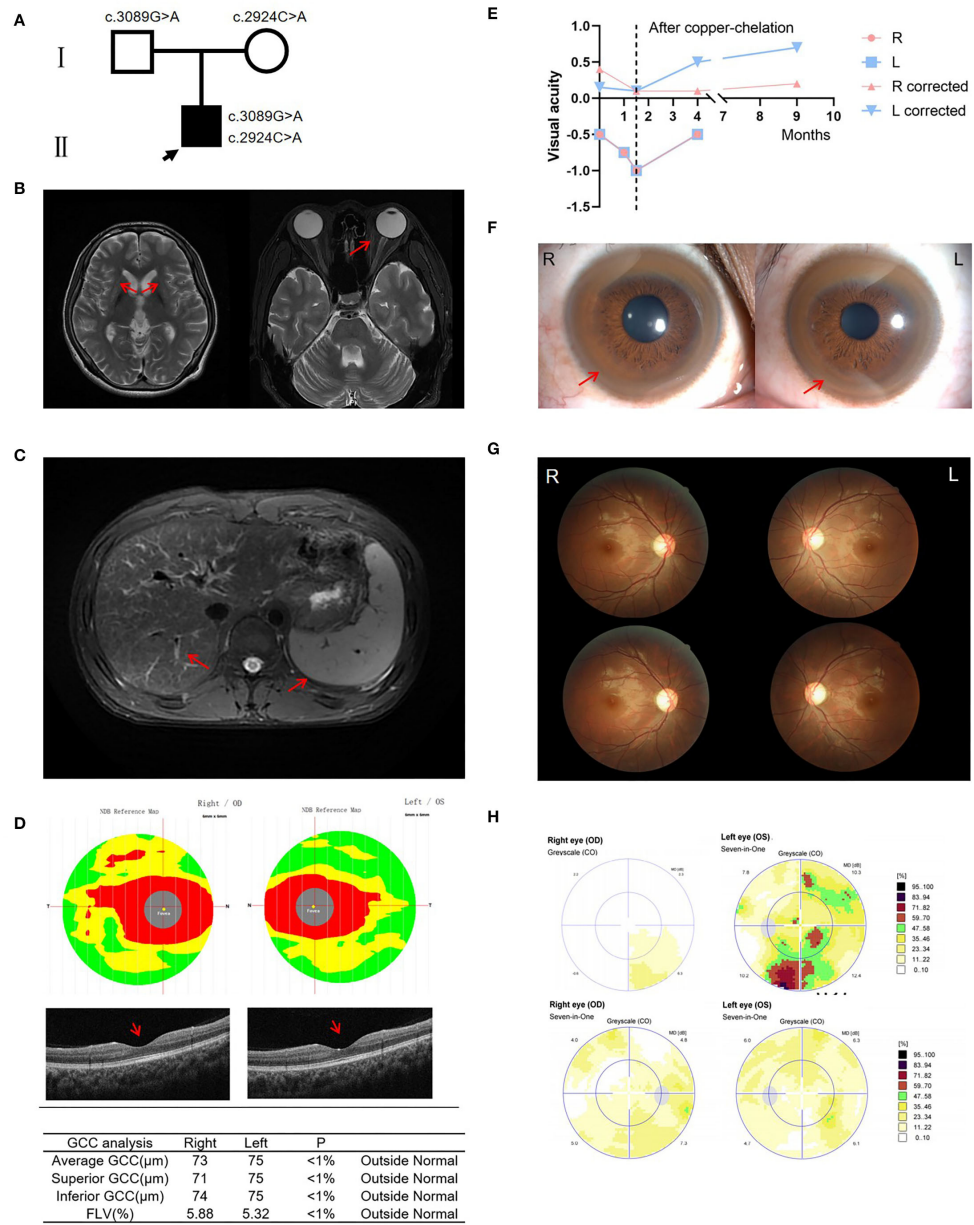
Clinical information of the patient. **(A)** Pedigree of the family. **(B)** Brain MRI showed symmetric hyperintense changes visualized in T2-weighted images of the basal ganglia, particularly the putamen; Orbit MRI showed no evidence of signal abnormality involving optic nerve or external compressive mass lesion. **(C)** Hepatic MRI revealed liver cirrhosis and splenomegaly. **(D)** OCT showed reduced thickness of the ganglion cell complex (GCC) and retinal nerve fiber layer (RNFL), and significantly damaged papillo-macular bundle before treatment. **(E)** Three ophthalmic examinations prior to treatment revealed declining vision. After copper-chelation therapy, visual acuity increased significantly. **(F)** Kayser-Fleischer rings. **(G)** Fundus examinations showed that the temporal side of the optic disc is pale before treatment. Fundus examinations show improvement of the optic disc after copper-chelation therapy. **(H)** Automated visual field upon presentation, which showed a slow light response and pallor on the temporal side of the optic disc. 3 months later, improvement in visual field is noted. OCT, optical coherence tomography; GCC, ganglion cell complex; FLV, focal loss volume.

In August 2021, a 22-year-old Chinese male was first admitted to our department with acute progressive binocular vision blurring. He had been experiencing kinetic tremors, dysarthria, and personality change since 2017 without any treatment. He complained of 4-week binocular vision blurring associated with progressive slow reaction, unsteady walking, executive dysfunction, and declined mental status. During this time, he had been admitted to several ophthalmology hospitals, K-F rings were noted on slit-lamp examination (Figure 1F). Additionally, the serum level of ceruloplasmin was low at 8.19 mg/dl (22–58 mg/dl).

The patient presented with photophobia or tearing but denied pain with eye movements. He smoked 10–20 cigarettes per day but denied anti-obesity, alcohol, herbals, or illicit drug use before entering our hospital; he had no other risk factors for liver or eye disease, and no known family history of any ophthalmologic, neurologic, or hepatic diseases.

On admission, his physical examination revealed dysarthria, gait disturbance, and altered mental status. The patient had no scleral icterus, rash, pedal edema, asterixis, or focal neurologic deficits. Brain magnetic resonance imaging (MRI) showed symmetric hyperintense changes visualized in T2-weighted images of the basal ganglia, particularly the putamen. However, orbit MRI showed no evidence of abnormality involving optic nerves or external compressive mass lesions (Figure 1B). Hepatic MRI revealed liver cirrhosis and splenomegaly (Figure 1C). Optical coherence tomography (OCT) showed reduced thickness of the ganglion cell complex (GCC) and retinal nerve fiber layer (RNFL), and significantly damaged papillomacular bundle (Figure 1D). Three ophthalmic examinations prior to treatment revealed declining vision: first time: right eye −0.5, corrected visual acuity 0.4; left eye −0.5, corrected visual acuity 0.15; second time: right eye −0.75; left eye −0.75; third time: right eye −1, corrected visual acuity .1; left eye −1, corrected visual acuity 0.1 (Figure 1E). Fundus examinations showed a slow light response and pallor in the temporal side of the optic disc (Figure 1G). Static perimetry revealed diffuse visual field defects in the left eye (Figure 1H). An ophthalmic diagnosis of optic neuropathy was made. Laboratory tests revealed prolonged PT/INR and APTT, and decreased ceruloplasmin at 54 mg/L (200-600 mg/L). 24-h urinary copper excretion was 117.5 μg/24 h (15–60 μg/24 h).

As K-F ring and low ceruloplasmin are characteristic of WD, he underwent a genetic test and was confirmed to be carrying *ATP7B* pathogenic variants in biallelic –c.3089G>A p.G1030D (paternal) and c.2924C>A p.S975Y (maternal) (Figure 1A). Meanwhile, considering other possible etiologies, a central nervous system demyelination laboratory test was taken, and AQP4-IgG, MOG-IgG, MBP-IgG, and GFAP-IgG were all negative. The most common mutations of Leber's hereditary optic neuropathy (LHON), 11778, 14484, and 3460, were also negative.

During a 3-time hospitalization in 9 months, he was given sodium dimercaptopropane sulfonate (DMPS) through intravenous infusion for copper-chelation, zinc gluconate to inhibit the absorption of copper, folic acid and mecobalamin to nourish the nerves, and symptomatic treatments such as improving mood with Citalopram Hydrobromide tablets (17). After a regular low-copper diet and D-penicillamine 125 mg tid intake at home, the symptoms of unsteady walking, blurred vision, altered mental status, slurred speech, and other symptoms have improved compared with the previous. 24-h urinary copper excretion was 2,332.4 μg/24 h (after copper-chelation), and liver enzyme levels become normal, demonstrating copper-chelation therapy was effective. Besides, the perimetry examination showed that the visual field has improved compared with 9 months before (Figure 1E). Fundus examinations were also better, with no visible disc edema, pallor, or maculopathy (Figure 1G). Automated visual fields presented significant improvement after the copper-chelation treatment (Figure 1H). Interestingly, the ophthalmic examination revealed that the visual acuity improved: right eye – 0.5, corrected visual acuity 0.1; left eye – 0.5, corrected visual acuity 0.5 (4 months later); right corrected visual acuity 0.2, left corrected visual acuity 0.7 (9 months later). The improvement in manifestations suggested that WD may be the etiology.

### Literature Review

WD is first described by Kinnear Wilson (18). Patients have variable clinical manifestations and laboratory test results, resulting in diagnostic dilemmas (17). The ophthalmological manifestations of WD include the typical K-F ring and an infrequent sign of sunflower cataract. No case of untreated Wilson's disease with visual impairment has been described until 1922, and Soderbergh described a 35-year-old farmer with bilateral optic atrophy in association with dysphagia, gait disturbance, and cirrhosis (12). In 1991, Rossa V. described a case of a 22-year-old male patient with WD who presented with retinal changes (13). In 2001, Gow PJ reported the case of a 46-year-old man who presented with end-stage liver disease caused by WD and who had associated rapidly progressive optic neuropathy (14). In 2013, Rukunuzzaman M. presented a 14-year-old boy with WD and blindness, tremor, and slurred speech along with end-stage liver disease (15). The blindness was thought to be due to optic neuropathy, which was reversed after drug treatment. In 2019, Liyung Tiffany Chou described a 20-year-old female patient with WD who presented with acute liver failure and associated monocular vision loss (16).

In general, patients with WD who have severe hepatic and/or neuropsychiatric symptoms without treatment are likely to develop acute-onset visual impairment. Improvement was noted after copper-chelation therapy.

## Discussion

Recently, some studies have shown that the optic nerve and retina may be affected in WD (19). Thinning of RNFL and macular thickness (Mth) examined by OCT was observed in patients with WD, especially in those with marked brain damage (20). Besides, degeneration of the retina in WD may serve as a marker of neurodegeneration and correlate with the degree of impairment of the nervous system (21).

The diagnosis of visual impairment in our patient was confirmed by the fundus examination, perimetry examination, and OCT. The patient had ingested no drugs before the manifestations, and he was excluded for multiple sclerosis and vasculitis. Mitochondria DNA screening for Leber's hereditary optic neuropathy was intact. Ultimately, the affected individual's diagnosis was established as WD, and the ocular manifestation was correlated with WD. In addition, after the low-copper diet and regular copper-chelation therapy, the symptoms improved compared with the previous. Inevitably, this raises questions about WD and its relationship with visual impairment, especially in light of the similar reports mentioned previously.

WD is a copper overload disorder that can potentially result in secondary hemochromatosis of iron overload. The pathophysiological mechanism involves reduced ceruloplasmin (22). As mentioned, ceruloplasmin plays a vital role in copper mobilization and secretion. Besides, ceruloplasmin is a serum ferroxidase functioning in iron turnover from oxidation of Fe^2+^ to Fe^3+^, a process which is essential for iron-binding to transferrin (the main iron-transporting protein) (23). It has been found that iron overload can cause optic nerve atrophy, strabismus, nystagmus, retinal degeneration, or night-blindness (24, 25). Thus, visual impairment in patients with WD can be expected to be due to impaired copper as well as iron metabolism. However, a recent study found that anti-copper treatment improved but did not normalize iron metabolism in WD (26). The improvement after treatment further suggested that copper rather than iron deposition may be the etiology. Still, the exact mechanism of how copper-chelation drugs recover vision remains unclear. Further studies with larger sample sizes of patients are needed to confirm the association between WD and visual impairment. Also, animal models are needed to clarify its mechanisms.

In addition, D-penicillamine may also cause visual impairment. Four cases of visual impairment complicated by penicillamine treatment have been reported in patients with WD (27–30). In these cases, the manifestation was corrected after discontinuation of penicillamine and administration of pyridoxine. Since our patient was not treated with D-penicillamine prior to the genetic diagnosis of WD in our institution, his decreased vision was unlikely to be drug-induced.

In conclusion, an acute-onset visual impairment associated with WD has rarely been described in the past. The circumstances of this case suggest that WD may be a rare cause of visual impairment, and that there is a potential association between visual impairment and WD. While the etiology behind patients' acute-onset visual impairment remains uncertain, the neuropsychiatric and hepatic symptoms, corneal K-F ring, decreased serum ceruloplasmin, and lower likelihood or exclusion of other causes raise the possibility of WD. Moreover, it is also vital to remain open to the optical manifestation of WD beyond K-F rings and sunflower cataracts and to act with sensitivity and caution when caring for patients with WD who may have subclinical ocular diseases. Clinicians need to recognize this rare manifestation and give appropriate treatment to avoid misdiagnosis and unnecessary overtreatment.

## Data Availability Statement

The original contributions presented in the study are included in the article/supplementary material, further inquiries can be directed to the corresponding author/s.

## Ethics Statement

The studies involving human participants were reviewed and approved by Second Affiliated Hospital of Zhejiang University School of Medicine. The patients/participants provided their written informed consent to participate in this study.

## Author Contributions

Z-WZ and M-HX designed the concept of this study and drafted the manuscript. C-BS, Z-YW, and YD critically reviewed the manuscript. All authors have read and approved the final manuscript.

## Funding

This study was supported by the grant (81701126) to YD from the National Natural Science Foundation of China.

## Conflict of Interest

The authors declare that the research was conducted in the absence of any commercial or financial relationships that could be construed as a potential conflict of interest.

## Publisher's Note

All claims expressed in this article are solely those of the authors and do not necessarily represent those of their affiliated organizations, or those of the publisher, the editors and the reviewers. Any product that may be evaluated in this article, or claim that may be made by its manufacturer, is not guaranteed or endorsed by the publisher.

## Abbreviations

WD: Wilson's disease
K-F ring: Kayser-Fleischer ring
MRI: magnetic resonance imaging
INR: international normalized ratio
PT: prothrombin time
APTT: activated partial thromboplastin time
β-CTx: β-crosslaps
PICP: propeptide of typeIprocollagen
LHON: Leber's hereditary optic neuropathy
MS: mean sensitivity
MD: mean defect
OCT: optical coherence tomography
GCC: ganglion cell complex
FLV: focal loss volume
RNFL: retinal nerve fiber layer
Mth: macular thickness
UWDRS: Unified Wilson's Disease Rating Scale.
